# Semaglutide-associated kidney injury

**DOI:** 10.1093/ckj/sfae250

**Published:** 2024-08-13

**Authors:** Farhana Begum, Kelly Chang, Krishna Kapoor, Rajiv Vij, Gautam Phadke, Wesley M Hiser, Rimda Wanchoo, Purva Sharma, Nirja Sutaria, Kenar D Jhaveri

**Affiliations:** Northwell Health, New Hyde Park, NY and Department of Medicine, Donald and Barbara Zucker School of Medicine at Hofstra/Northwell, Manhasset, NY, USA; Department of Internal Medicine, Christus Health/Texas A&M School of Medicine, Longview, TX, USA; Department of Internal Medicine, Christus Health/Texas A&M School of Medicine, Longview, TX, USA; Department of Internal Medicine, Christus Health/Texas A&M School of Medicine, Longview, TX, USA; Metrolina Nephrology Associates; Atrium Health, Charlotte, NC, USA; Baylor Scott and White Medical Center, Pathologists Bio-Medical Laboratories, Dallas, TX, USA; Northwell Health, New Hyde Park, NY and Department of Medicine, Donald and Barbara Zucker School of Medicine at Hofstra/Northwell, Manhasset, NY, USA; Division of Kidney Diseases and Hypertension, Donald and Barbara Zucker School of Medicine at Hofstra/Northwell, Great Neck, NY, USA; Northwell Health, New Hyde Park, NY and Department of Medicine, Donald and Barbara Zucker School of Medicine at Hofstra/Northwell, Manhasset, NY, USA; Division of Kidney Diseases and Hypertension, Donald and Barbara Zucker School of Medicine at Hofstra/Northwell, Great Neck, NY, USA; Department of Internal Medicine, Atrium Health Wake Forest University School of Medicine, Charlotte, NC, USA; Northwell Health, New Hyde Park, NY and Department of Medicine, Donald and Barbara Zucker School of Medicine at Hofstra/Northwell, Manhasset, NY, USA; Division of Kidney Diseases and Hypertension, Donald and Barbara Zucker School of Medicine at Hofstra/Northwell, Great Neck, NY, USA

**Keywords:** acute interstitial nephritis, AIN, AKI, diffuse podocytopathy, GLP1 agonist, minimal change disease, podocytopathy, semaglutide

## Abstract

Glucagon-like peptide-1 receptor agonists (GLP-1RAs) are multipurpose agents effective in improving glycemic control in patients with type 2 diabetes while also achieving weight loss and risk reduction of major cardiovascular (CV) events and chronic kidney disease progression. With their increased utility in diabetes, obesity, CV health and renal protection, the use of GLP-1RAs has increased. However, with this increased use, there have also been increased reports of associated kidney adverse events, including case reports of acute interstitial nephritis (AIN) associated with GLP-1RA use. We report the data from the Food and Drug Administration adverse event reporting system (FAERS) in relation to GLP-1RA use and adverse kidney events, with acute kidney injury being the most common. In addition, we report two cases of semaglutide-associated biopsy-proven AIN and one with associated podocytopathy. To our knowledge, this is the first case of biopsy-proven AIN with podocytopathy associated with semaglutide use. Both patients experienced complete remission shortly after discontinuing semaglutide and undergoing immunosuppressive therapy. Further analysis of the FAERS database revealed 17 cases of proteinuria and 1 case of glomerulonephritis associated with semaglutide in the FAERS database, however no further information was available. While further research is needed to establish causality, this case series adds to the growing body of literature that semaglutide is associated with AIN and adds a new association, semaglutide with AIN and podocytopathies. While the overall clinical and mortality benefits of GLP-1RAs may outweigh the rarer risks, prescribers need to be aware of these associations, particularly as the use of GLP-1RAs continues to expand.

## INTRODUCTION

Glucagon-like peptide-1 receptor agonists (GLP-1RAs) are incretin analogues that potentiate glucose-dependent insulin excretion, delay gastric emptying, suppress glucagon secretion and reduce appetite [1]. They are multipurpose agents that not only are effective in improving glycaemic control in patients with type 2 diabetes, but have also revolutionized treatment for achieving weight loss and risk reduction of major cardiovascular (CV) events and chronic kidney disease (CKD) progression [2]. Semaglutide is a GLP-1RA dosed weekly that is Food and Drug Administration (FDA)-approved for obesity after results from the semaglutide treatment effect in people with obesity (STEP) trials showed reduced body weight in non-diabetic patients with this agent [3]. Semaglutide was also found to reduce CV-related death, nonfatal MI and nonfatal strokes in diabetic patients with the trial to evaluate cardiovascular and other long-term outcomes with semaglutide in subjects with type 2 diabetes (SUSTAIN-6) trials, and later in non-diabetic obese patients with the semaglutide effects on cardiovascular outcomes in people with overweight or obesity (SELECT) trial [2, 4]. More recently, the FLOW trial studied semaglutide in patients with diabetes and CKD and was terminated early based on remarkable efficacy in delaying progression of CKD [5]. With their utility in diabetes, obesity, CV health and kidney protection, the use of GLP-1RAs has increased in recent times. However, with this increased use, there have also been increased reports of associated adverse events.

A review completed in 2014 quantified the adverse events related to GLP-1RA use [6]. The most common symptoms reported were gastrointestinal, mainly nausea. Several (>100) case reports did link the use of GLP-1RAs with acute kidney injury (AKI), mainly seen with exenatide use, and deemed primarily secondary to pre-renal azotemia from nausea, vomiting and diarrhea (Filippatos *et al*. [6]). Exenatide-associated acute interstitial nephritis (AIN) in CKD patients, which improved with steroid use, was reported but cases were uncommon [6, 7]. However, since this 2014 review, due to the studies mentioned above, GLP-1RAs use, particularly semaglutide use, has increased and therefore more kidney-related adverse events have been noted.

In this report, we summarize two cases of biopsy-proven AIN and one with concomitant focal segmental glomerulosclerosis (FSGS), a podocytopathy in association with semaglutide use. Additionally, we report the data from the FDA adverse event reporting system (FAERS) regarding GLP-1RA use to better assess kidney-related adverse events from GLP-1RAs use.

## CASE REPORTS

### Case 1

**Figure 1:**
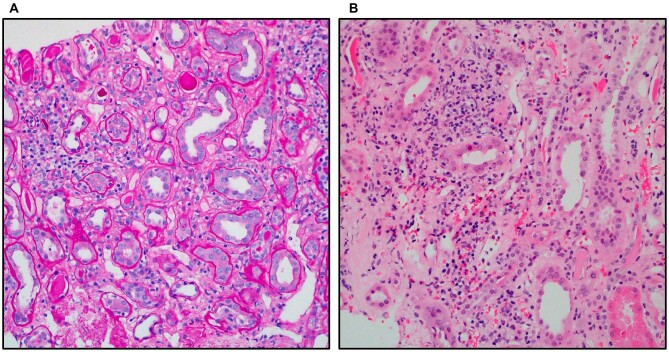
From Case 1. Image (**A**) periodic acid–Schiff (PAS) Patchy infiltrates with associated tubulitis and tubular injury characterized by epithelial flattening and attenuated brush borders (PAS ×200). Image (**B**) hematoxylin and eosin (H&E) 20, H&E 40: infiltrates comprising of mononuclear inflammatory cells with scattered eosinophils. There is evidence of acute tubular injury with flattening of epithelial cells and focal mild tubulitis (H&E ×200; H&E ×400).

A 68-year-old female presented to the emergency department due to a serum creatinine (SCr) of 6.72 mg/dL (baseline 1.2–1.5 mg/dL) detected during a routine 4-month laboratory examination. After the initiation of weekly semaglutide 0.25 mg injections 3 weeks prior, she began experiencing nausea and vomiting that resolved a few days before presentation. Her past medical history included CKD stage IIIa, type II diabetes, hypertension, hyperlipidemia, coronary artery disease, hypothyroidism, gastroesophageal reflux disease and class II obesity. Chronic medications included aspirin, atorvastatin, carvedilol, escitalopram, ferrous sulfate, furosemide, insulin, levothyroxine, lisinopril, intrathecal morphine, pantoprazole and trazodone. Her exam noted a blood pressure of 150/71 mmHg and mildly delayed capillary refill. Laboratory testing revealed SCr of 6.31 mg/dL and serum blood urea nitrogen (BUN) of 46 mg/dL. Urinalysis revealed a specific gravity of 1.010, proteinuria (1+), glucosuria, hyaline casts, trace leukocyte esterase and trace blood. Fractional excretion of sodium was 2.5%. She was started on a normal saline infusion. Kidney ultrasound demonstrated mild bilateral cortical atrophy with no hydronephrosis. Furosemide, lisinopril and semaglutide were discontinued. Pantoprazole was continued throughout the admission and at discharge. SCr improved to 3.07 mg/dL after 9 days of hospitalization. Within a week of discharge, she resumed lisinopril and received one dose of semaglutide. Laboratory examination 2 weeks later revealed a rise in SCr to 4.1 mg/dL, which up-trended to 4.84 mg/dL 2 weeks later. She was instructed to discontinue lisinopril, pantoprazole and semaglutide, and return to the hospital. Laboratory studies in the hospital showed SCr of 5.1 mg/dL and serum BUN of 34 mg/dL. Urinalysis showed a specific gravity of <1.005, proteinuria (1+), glucosuria, trace blood and few white blood cells. The patient was hydrated with a saline-based infusion. After a 1-week admission, SCr improved to 3.26 mg/dL. A kidney biopsy 1 month after discharge showed scattered eosinophils and tubilits suggestive of acute mild tubulointerstitial nephritis as well as diffuse diabetic glomerulosclerosis, and moderate arteriosclerosis (Fig. 1). She was started on prednisone 60 mg daily which was gradually tapered over 3 months, with improvement in the SCr to 1.5 mg/dL. The patient was started on linagliptin and omeprazole while on steroid therapy. The patient was not re-challenged with another GLP-1RA. The most recent SCr was 2.0 mg/dL, 2 months after discontinuing steroids. It was concluded that this was a case of AIN linked to semaglutide use in the setting of CKD from diabetic nephropathy.

### Case 2

A 49-year-old African-American female presented to the emergency room with 8 weeks of bilateral pedal edema, and 4.99 kg of weight gain. Three months prior to presentation, the patient was started on semaglutide 0.5 mg weekly for weight loss. Her past medical history included morbid obesity (body mass index 44 kg/m^2^) and controlled hypertension. She was on amlodipine 10 mg and valsartan 320 mg daily. No non-steroidal anti-inflammatory drug use was noted. On presentation to the emergency department, her labs were notable for a SCr of 1.6 mg/dL (baseline SCr was 0.9–1.2 mg/dL), hypoalbuminemia of 2.6 mg/dL, urinalysis with 10–25 red blood cells per high-power field, no pyuria and a urine protein–creatinine ratio of 11 g/g. Eighteen months prior, her baseline proteinuria was 0.4 mg/mg. HIV, hepatitis panel, anti-MPO antibody, anti-PR3 antibody, PLA2R antibody, SPEP, immunofixation and free light chains were all negative. Given the new worsening, severe proteinuria, and worsening kidney injury, the patient underwent a kidney biopsy. The kidney biopsy revealed FSGS, not otherwise specified, and AIN with mild interstitial fibrosis and tubular atrophy. Ultrastructural examination revealed glomerular basement membranes with variable thickening of the lamina densa, with an associated segmental effacement (25%) of the overlying foot processes, including short segments of podocyte dropout with denudation of the underlying basement membrane and microvillous transformation of the podocyte's cytoplasm. No immune complex–type electron dense deposits were identified. Given the temporal relationship between initiating semaglutide and kidney injury, AIN and podocytopathy were linked to the use of this medication and semaglutide was discontinued. Genetic testing was not performed. The patient was treated with 0.5 mg/kg prednisone for 4 weeks, with dose-tapering by 10 mg every 2 weeks. Twelve weeks post-treatment and post-discontinuation of semaglutide, the patient's SCr improved to 1.3 mg/dL with a urine protein–creatinine ratio of 0.6 g/g. The patient started on valsartan 320 mg daily and semaglutide was never resumed.

### FAERS data review

Given the increase in GLP-1RA use, we reviewed the FAERS and queried for kidney adverse events associated with GLP-1RAs (semaglutide, exenatide, tirzepatide, liraglutide, albiglutide, dulaglutide) between 2010 and 2022 using search terms all related to and including acute kidney injury, AKI, electrolyte disorders, and hypertension. SAS enterprise guide v7.12 was used to pull data.

In the last 12 years, a total of 2375 total kidney-related adverse events were reported to the FAERS in reference to GLP-1RAs. AKI (58.65%) was the most common, with equal distribution for males vs females. Of note, there were 17 cases of proteinuria and 1 case of glomerulonephritis associated with semaglutide in the data. Hypertension (22.02%) was the next most common event, followed by hyperkalemia as the most common electrolyte disorder. Most of the other electrolyte disorders and capillary leak syndrome were uncommon (Table 1). It appears nephrotic-range proteinuria and/or podocytopathies was a rare finding even amongst the total 2375 kidney-related events.

**Table 1:** FAERS data review of GLP-1RAs-associated renal injury (semaglutide, exenatide, tirzepatide, liraglutide, albiglutide, dulaglutide).

Name of		Male	Female	Missing	Overall
medication	Reaction	(*N* = 1023), *n* (%)	(*N* = 1160), *n* (%)	(*N* = 192), *n* (%)	(*N* = 2375), *n* (%)
GLP-1RAs	Renal injury	586 (57.28)	681 (58.71)	126 (65.63)	1393 (58.65)
	Hypertension	212 (20.72)	279 (24.05)	32 (16.67)	523 (22.02)
	Hyperkalaemia	64 (6.26)	22 (1.90)	9 (4.69)	95 (4.00)
	Hypokalemia	35 (3.42)	45 (3.88)	9 (4.69)	89 (3.75)
	Thrombocytopenia	34 (3.32)	37 (3.19)	0 (0.00)	71 (2.99)
	Hyponatremia	22 (2.15)	24 (2.07)	3 (1.56)	49 (2.06)
	Proteinuria	17 (1.66)	11 (0.95)	2 (1.04)	30 (1.26)
	Acidosis	14 (1.37)	16 (1.38)	2 (1.04)	32 (1.35)
	Hypercalcemia	13 (1.27)	12 (1.03)	1 (0.52)	26 (1.09)
	Hypomagnesemia	11 (1.08)	17 (1.47)	2 (1.04)	30 (1.26)
	Hypocalcemia	5 (0.49)	5 (0.43)	1 (0.52)	11 (0.46)
	Hypophosphatemia	4 (0.39)	7 (0.60)	2 (1.04)	13 (0.55)
	Hypernatremia	2 (0.20)	2 (0.17)	2 (1.04)	6 (0.25)
	Renal tubular acidosis	2 (0.20)	0 (0.00)	0 (0.00)	2 (0.08)
	Tumour lysis syndrome	1 (0.10)	1 (0.09)	0 (0.00)	2 (0.08)
	Hypertensive urgency	1 (0.10)	0 (0.00)	0 (0.00)	1 (0.04)
	Hyperphosphatemia	0 (0.00)	1 (0.09)	0 (0.00)	1 (0.04)
	Capillary leak syndrome	0 (0.00)	0 (0.00)	1 (0.52)	1 (0.04)

Adverse events reported as renal failure, renal impairment, renal failure acute, renal injury, nephritis, presented as one group (renal injury).

Percentage (%) = *n*/(*N* * 100).

The events are reported by providers and/or patients and therefore could have a reporting bias. Most importantly, it is not possible to determine whether an event is truly caused by the drug as opposed to the underlying disease, concomitant medications or by prior drugs administered to these patients. In addition, we cannot get an accurate assessment of the incidence rate, as we do not have complete information on the total number of patients who have received these agents.

However, there are important limitations that one must keep in mind when using the FAERS database. The events are reported by providers and/or patients and therefore could have a reporting bias. Most importantly, it is not possible to determine causality from the drug as opposed to adverse events from the underlying disease, concomitant medications or by prior drugs administered to these patients. In addition, we cannot get an accurate assessment of incidence rate, as we do not have complete information on the total number of patients who have received these agents.

## DISCUSSION

Since 2015, as GLP-1RAs gained popularity, more cases of GLP-1RA-associated AIN have been reported (Table 2). Leehey *et al*. reported two cases of AKI on CKD with semaglutide use that showed AIN on kidney biopsy where kidney function did not improve with cessation of semaglutide use in January 2021 [8]. A literature search published in December 2021 reported six cases of AIN associated with GLP-1RAs in patients with CKD, particularly with liraglutide (one), exenatide (three), semaglutide (one) and dulaglutide (one). Only two patients had full recovery of kidney function, neither of which were treated with steroids [9]. A 2021 case report reported the first semaglutide-induced AIN in a patient with no background CKD [10]. The first case of biopsy-proven dulaglutide associated with AIN was reported in October 2022 [11]. Of note in this case, the patient did not have CKD and was on exenatide for many years before being switched to dulaglutide which caused AKI 4 weeks later [11]. This raises the question of whether some GLP-1RAs are more immunogenic than others and whether some patients should be re-challenged with a different GLP-1RAs given its proven cardiovascular and renal benefits. As can be seen from Table 2, most reported cases ranged from ages 60 to 80 years, evenly distributed between males and females, and most patients had a history of CKD prior to the kidney injury. Table 2 also highlights that most patients given steroids had recovery of kidney function, while there were mixed results to only stopping the offending agent—some recovered, while others did not. As the use of GLP-1RAs increases, we suspect that there will be an associated increase in AIN reporting.

**Table 2:** Published cases of GLP-1RA-induced AIN and MCD.

Drug name	Author	Age/sex	Initial renal function	Time to presentation	Renal function post-GLP-1	Biopsy/diagnosis	Treatment	Recovery
AIN								
Liraglutide	Gariani *et al*. 2014 [16]	83M	Creatinine 2.14 mg/dL; eGFR 32 mL/min/1.73 m^2^		Creatinine 9.3 mg/dL; eGFR 6 mL/min/1.73 m^2^	Confirmed/AIN	Stopped liraglutide; steroids and transient dialysis	Partial recovery
Exenatide	Dubois-Laforgue *et al*. 2014 [17]	75M	Creatinine 130 μmol/L	5 days	Creatinine 1148 μmol/L	Not done/AIN	Stopped exenatide; haemodialysis for 48 h insulin therapy	Full recovery, 9 days
Exenatide	Bhatti *et al*. 2010 [18]	65F	Creatinine 77 μmol/L; eGFR 66 mL/min/1.73 m^2^	9 weeks	Creatinine 393 μmol/L; eGFR 10 mL/min/1.73 m^2^	Not done/AIN	Exenatide stopped; prednisolone	Partial recovery, 6 weeks
Exenatide	Nandakoban *et al*. 2013 [7]	58M	Creatinine 120 μmol/L; eGFR 59 mL/min/1.73 m^2^	2 months	SCr 209 μmol/L; eGFR 39 mL/min/1.73 m^2^	Confirmed/AIN	Stopped exenatide; prednisolone	Partial recovery, 4 months
Semaglutide	Leehey *et al*. 2021 [8]	∼80F	Creatinine 1.59 mg/dL; eGFR 30 mL/min/1.73 m^2^	5 months	Creatinine 3.50 mg/dL; eGFR 11 mL/min/1.73 m^2^	Confirmed/AIN	Discontinued semaglutide	No recovery
Dulaglutide	Taylor *et al*. 2018 [19]	63F	Creatinine 1.6 mg/dL; eGFR 34 mL/min/1.73 m^2^	1 month	Creatinine 3.4 mg/dL; eGFR 13.7 mL/min/1.73 m^2^	Not done/AIN	Discontinued dulaglutide	Full recovery, 4 weeks
Semaglutide	Borkum *et al*. 2022 [10]	∼30M	Creatinine 1.05 mg/dL; eGFR 91 mL/min/1.73 m^2^	6 weeks	Creatinine 12.86 mg/dL	Confirmed/AIN	Discontinued semaglutide; prednisone	Full recovery, 4 weeks
Dulaglutide	Komala *et al*. 2022 [11]	78M	Normal	4 weeks	Creatinine 8.46 mg/dL	Confirmed/AIN	Discontinued dulaglutide; prednisolone	Full recovery, 2 weeks
Liraglutide	Chaudhury *et al*. 2021 [9]	59F	eGFR 35 mL/min/1.73 m^2^	5 months	eGFR 17 mL/min/1.73 m^2^	Confirmed/AIN	Discontinued liraglutide; on peritoneal dialysis	No recovery
Tirzepatide	Espino *et al*. 2023 [20]	42F	Creatinine 3.23 mg/dL; eGFR 16 mL/min/1.73 m^2^	1 month	Creatinine 0.49 mg/dL; eGFR 121 mL/min/1.73 m^2^	Not done/AIN	Discontinued tirzepatide	Recovering, days
Podocytopathies								
Semaglutide	Attieh *et al*. 2024 [12]	43F	Proteinuria normal; creatinine 0.9–1.1 mg/dL	12 weeks	UACR 10.27 g/g; creatinine 1.34 mg/dL	Confirmed/MCD	Oral prednisone; discontinued semaglutide 5 weeks later	Full recovery 2 weeks post-semaglutide discontinuation
Semaglutide	Attieh *et al*. 2024 [12]	39M		6 months	UPCR 9.7 g/g; creatinine 1 mg/dL	Confirmed/MCD	Semaglutide discontinued; rituximab	Full recovery in 1 month
Semaglutide	Attieh *et al*. 2024 [12]	60F		10 weeks	UACR 2 g/g; creatinine 0.64 mg/dL	Confirmed/MCD	Semaglutide discontinued; prednisone	Full recovery in 2 months

eGFR, estimated glomerular filtration rate; UACR, urine albumin to creatinine ratio; UPCR, urine protein to creatinine ratio.

In Case 1, the diagnosis of semaglutide-induced AIN was supported by biopsy findings of scattered eosinophils and tubulitis as well as renal impairment that worsened and improved following semaglutide initiation and discontinuation, respectively, on two separate occasions. Although the patient had been on other known offending agents, including aspirin, furosemide and particularly pantoprazole, as proton-pump inhibitors (PPIs) are one of the most common causes of drug-induced AIN, the patient's SCr had previously been stable on these chronic medications, and did not up-trend with its resumption. In Case 2, the patient's SCr increased from 1.2 to 1.6 mg/dL with nephrotic-range proteinuria approximately 4 weeks after semaglutide initiation. The creatinine and proteinuria both improved after semaglutide discontinuation and steroid treatment. AIN typically is not associated with nephrotic-range proteinuria. However, in Case 2, the patient's biopsy revealed AIN with FSGS or steroid response podocytopathy. In both cases, the patients had some stage of CKD prior to the AKI, like other cases reported in the literature. Recently, we reported a case series of three cases of semaglutide-associated podocytopathies (anti-nephrin associated). Semaglutide induces an anti-nephrin-related injury on the podocyte, highlighting the role of GLP-1RAs in immune system modulation, and suggesting their potential involvement in triggering autoimmunity [12]. To our knowledge Case 2 is the first reported case of AIN with FSGS seen in association with GLP-1RA use. Mechanistically, however, the two disease processes may be connected. One possible mechanism for how GLP-1RAs can cause podocytopathies such as FSGS and minimal change disease (MCD) is that GLP-1RAs are peptides and thus possess immunogenic potential that may cause podocytopathy via a type 2 systemic hypersensitivity reaction like drug-induced AIN [13]. Our patient responded to steroid therapy, which could have improved the AIN and the podocytopathy.

To add to this literature, we also reviewed the FAERS and queried for kidney adverse events associated with GLP-1RA, and found that from 2010 to 2022 renal injury (AKI) was reported in 586 patients and was the most common kidney adverse event. However, current evidence suggests that GLP-1RAs do not increase the overall risk of AKI at a population level [14, 15]. Some studies even suggest that these medications may reduce the progression of nephropathy in patients with diabetes, as can be seen from the evaluate renal function with semaglutide once weekly (FLOW) trial [2, 14, 15]. Given the proteinuria and podocytopathy seen in Case 2, we identified 17 cases of proteinuria out of 2375 reports, and 1 case of glomerulonephritis associated with semaglutide in the FAERS database, which appears to show a low prevalence. However, there are important limitations that one must keep in mind when using the FAERS database as previously mentioned, the most important being an inability to prove causation or incidence rate.

It is important to identify the risk factors for GLP-1RA-induced AIN to determine which patients need to be closely monitored during drug initiation. Based on previous reported cases (Table 2), most patients were elderly and had some history of CKD. Therefore, possible risk factors include CKD (as in Case 1), advanced age, obesity (as in Case 2) and concurrent administration of medications that cause AIN (PPI in Case 1) [15]. Further investigation is needed to determine whether some GLP-1RAs are more immunogenic than others, and whether certain patients would benefit from being re-challenged on a different GLP-1RA given its overall health benefits.

As GLP-1RAs are being used for type 2 diabetes, weight loss, CV and kidney protection, we need to be aware of AKI and glomerulopathy as potential adverse events associated with this class of agents. Although the overall mortality, CV and renal benefits of this class of drugs are clinically significant, our cases emphasize the need to identify whether GLP-1RAs can cause renal dysfunction in some patients, especially in the setting of CKD, both through volume depletion from known gastrointestinal side effects and through intrinsic mechanisms such as AIN and diffuse podocytopathies such as MCD and FSGS.
